# Diet-Induced Obesity Modulates Epigenetic Responses to Ionizing Radiation in Mice

**DOI:** 10.1371/journal.pone.0106277

**Published:** 2014-08-29

**Authors:** Guillaume Vares, Bing Wang, Hiroko Ishii-Ohba, Mitsuru Nenoi, Tetsuo Nakajima

**Affiliations:** Research Center for Radiation Protection, National Institute of Radiological Sciences, Inage-ku, Chiba, Japan; University of Dundee, United Kingdom

## Abstract

Both exposure to ionizing radiation and obesity have been associated with various pathologies including cancer. There is a crucial need in better understanding the interactions between ionizing radiation effects (especially at low doses) and other risk factors, such as obesity. In order to evaluate radiation responses in obese animals, C3H and C57BL/6J mice fed a control normal fat or a high fat (HF) diet were exposed to fractionated doses of X-rays (0.75 Gy ×4). Bone marrow micronucleus assays did not suggest a modulation of radiation-induced genotoxicity by HF diet. Using MSP, we observed that the promoters of *p16* and *Dapk* genes were methylated in the livers of C57BL/6J mice fed a HF diet (irradiated and non-irradiated); *Mgmt* promoter was methylated in irradiated and/or HF diet-fed mice. In addition, methylation PCR arrays identified *Ep300* and *Socs1* (whose promoters exhibited higher methylation levels in non-irradiated HF diet-fed mice) as potential targets for further studies. We then compared microRNA regulations after radiation exposure in the livers of C57BL/6J mice fed a normal or an HF diet, using microRNA arrays. Interestingly, radiation-triggered microRNA regulations observed in normal mice were not observed in obese mice. miR-466e was upregulated in non-irradiated obese mice. *In vitro* free fatty acid (palmitic acid, oleic acid) administration sensitized AML12 mouse liver cells to ionizing radiation, but the inhibition of miR-466e counteracted this radio-sensitization, suggesting that the modulation of radiation responses by diet-induced obesity might involve miR-466e expression. All together, our results suggested the existence of dietary effects on radiation responses (especially epigenetic regulations) in mice, possibly in relationship with obesity-induced chronic oxidative stress.

## Introduction

Over the last century, ample evidence has been accumulating of a strong link between carcinogenesis and exposure to ionizing radiation [1]. The deleterious effects of ionizing radiation are considered to result mainly from the direct induction of DNA damage in target cells, but also from non-targeted effects (such as radiation-induced genomic instability and bystander effect) [2]. Ionizing radiation can cause chromosomal aberrations, including acentric fragments, which upon cell replication are excluded from the main nuclei and form micronuclei (MN) [3]. The monitoring of MN in erythrocytes of the bone marrow is an effective tool to assess the genotoxic effects of ionizing radiation even at low levels. Radiation-induced genomic instability is a well-documented phenomenon, which can be observed even in the progeny of irradiated animals, suggesting the involvement of epigenetic mechanisms [4], such as the modulation of genome methylation or the regulation of micro-RNA expression. Recent data suggest that even low dose radiation exposures could also result in epigenetic modifications [5].

A major question for radiation biology is to understand how other risk factors could influence the biological effects of ionizing radiation, especially in terms of cancer risk [6], or reversely how ionizing radiation exposures could potentialize other risk factors. Obesity and associated disorders are increasingly becoming a global health challenge. Obesity is a major risk factor for various metabolic syndromes (such as insulin resistance, type 2 diabetes and nonalcoholic fatty liver disease) [7]–[9] and for initiation of cancer at several organ sites, including breast, endometrium and kidney [10]. Weight gain and obesity was also related to mortality from liver cancer, pancreatic cancer, non-Hodgkin lymphoma and myeloma [11]. Nonalcoholic fatty liver disease can evolve into nonalcoholic steatohepatitis, which can lead to primary hepatocellular carcinoma [12]. There is compelling evidence that oxidative stress and inflammation are major mechanisms involved in metabolic disorders associated with obesity [13].

The susceptibility to develop diet-induced obesity (DIO) and metabolic syndromes strongly depends on the genetic background, with heritability estimates ranging from 40% to 70% in human populations [14], [15]. Furthermore, a number of studies have shown that various mouse strains and their genetic variants show different metabolic phenotypes [16]. For example, the liver response to a sustained high-fat diet was characterized by the activation of peroxisomal β-oxidation in C57BL/6J mice and by lipogenesis in 129Sc mice [17]. While C57BL/6J mice exhibited severe obesity and diabetes after being fed a high-fat diet, C3H mice fed a similar diet showed normal glucose tolerance and no hepatic steatosis even though they exhibited a significant weight increase [18]. The C57BL/6J mouse strain has become a popular experimental model for evaluating the molecular mechanisms and consequences of DIO, because they develop insulin resistance, hyperglycemia and obesity in a way that closely matches the development of human metabolic syndrome [19]. In comparison, other strains, such as C3H/He, 129/Sv and A/J mice are more resistant to diabetes and obesity [20].

Emerging evidence suggests that dietary effects [21], as well as carcinogenesis [22], involve epigenetic mechanisms, such as DNA methylation changes or miRNA regulations. On the one hand, aberrant gene silencing through promoter hypermethylation has been shown to be a crucial event during early neoplastic progression [23]. High fat diet-induced obesity and metabolic syndromes can also modulate the methylation pattern of various gene promoters in human and animal models, such as leptin [24], peroxisome proliferator activated receptor γ (PPARγ) [25] or microsomal triglyceride transfer protein (*Mttp*) [26]. On the other hand, there is now ample (and still growing) data describing cancer-associated miRNA regulations, implying the crucial role of miRNAs in maintaining cellular homeostasis [27].

While radiation-induced carcinogenesis was significantly reduced in C3H mice after calorie restriction [28], there is a scarcity of data concerning the modulation of radiation responses by high fat diets or obesity. In a mouse model of malignant glioma, high fat low-carbohydrate ketogenic diet significantly enhanced the effectiveness of radiation therapy [29]. Obesity also increased non-ionizing UV radiation-induced oxidative stress [30].

In this study, we compared the short-term biological responses to ionizing radiation in C57BL/6J and C3H mice fed a control normal fat diet or a high fat (HF) diet. First, we evaluated whether DIO could influence radiation-induced genotoxicity. Then, because liver is one of the main target sites for metabolic syndrome, we measured immediate epigenetic regulations in relationship with diet and ionizing radiation (gene repression through promoter hypermethylation, miRNA regulations).

## Materials and Methods

### Mice

14 weeks-old C57BL/6J DIO mice and 5 weeks-old C3H/HeJ mice were purchased from Charles River Laboratories (Yokohama, Japan) and SLC, Inc. (Hamamatsu, Japan), respectively. C57BL/6J DIO mice are a model of pre-diabetic type 2 diabetes with elevated blood glucose and impaired glucose tolerance. C3H mice are HF diet-induced obesity resistant. Mice were maintained for 3 (C57BL/6J) or 12 weeks (C3H) in a conventional animal facility under a 12-h light/12-h dark photoperiod (lights on from 7∶00 a.m. to 7∶00 p.m.). Animals were housed in autoclaved cages with sterilized wood chips and allowed free access to 10% fat (D12450B, Research Diets Inc., New Brunswick, NJ, USA) or 60% fat laboratory chow (D12492, Research Diets Inc.) and acidified water (pH = 3.0±0.2). The 60% fat chow will be referred to as “high fat diet” (HF diet). All experimental protocols involving mice were reviewed and approved by *The Institutional Animal Care and Use Committee* of the National Institute of Radiological Sciences (NIRS) and were performed in accordance with the *NIRS Guidelines for the Care and Use of Laboratory Animals*.

### Cell culture and free fatty acid treatment

AML12 mouse liver cells (ATCC, CRL-2254) were grown in DMEM/F12 medium (Gibco, Gaithersburg, MD, USA) supplemented with 0.005 mg/ml insulin, 0.005 mg/ml transferrin, 5 ng/ml selenium, and 40 ng/ml dexamethasone (ITS Media Supplement, Sigma-Aldrich, St Louis, MO, USA) and 10% fetal bovine serum (FBS, Nichirei Biosciences, Tokyo, Japan). Oleic acid and palmitic acid (Sigma-Aldrich) were conjugated to bovine serum albumin at a molar ratio of 6∶1. Cells were treated with a mixture of oleic and palmitic acid for 24 hours before irradiation.

### Irradiation

Mice were exposed to four times daily doses of 0.75 Gy X-rays (0.25 Gy/min) generated by a Pantak 320S machine (Shimadzu, Japan) operated at 200 kVp and 20 mA, using a 0.50-mm Al +0.50-mm Cu filter. An exposure rate meter (AE-1321M, Applied Engineering Inc., Japan) was used for the dosimetry. Mice were sacrificed one day after the final irradiation.

AML12 cells were irradiated in serum-free medium 72 hours after plating, using an X-ray generator (ISOVOLT Titan-320, General Electric, Fairfield, CT, USA) at a dose-rate of 0.9 Gy/min.

### Bone marrow micronucleus test

Diet- and radiation-induced genotoxicity was assessed using the standard bone marrow MN test according to the method published by Hayashi *et al.* [31]. Bone marrow smears were prepared from both femurs. Micronuclei were counted in immature polychromatic erythrocytes (PCEs) and in mature normochromatic erythrocytes (NCEs). The frequencies of micronuclei in PCEs and NCEs are referred to as MNPCEs and MNNCEs, respectively. The ratio of PCEs to mature NCEs (P/N ratio) is an indicator of the relative proliferation rate in the erythroid lineage, and its decrease is considered to be an indicator of mutagen-induced cytotoxicity [32]. At least 15000 cells (PCE+NCE) per mouse were counted and the data for each experimental point were obtained from at least 5 mice.

### Flow cytometry

To identify cells of the erythroid lineage, bone marrow cells were incubated immediately after collection, with FITC-conjugated TER-119 antibody (557915, BD Pharmingen, San Diego, CA, USA) for 20 minutes at 4°C then washed, fixed with paraformaldehyde/methanol [33] and resuspended in cold PBS with 3% FBS, 50 µg/mL propidium iodide and 1 µg/mL RNase. The cell cycle repartition of erythroid cells was then analyzed on a FACSCalibur flow cytometer (Becton Dickinson, San Jose, CA, USA) with the CellQuest software (Becton Dickinson). Cell cycle analysis was performed using ModFit LT software (Verity Software House, Topsham, ME, USA).

### Methylation PCR array

For methylation analysis of mouse liver tissues, the EpiTect Methyl qPCR profiling service (Filgen, Nagoya, Japan) was used. Genomic DNA was extracted from C57BL/6J mouse livers using the QIAmp DNA Mini Kit (Qiagen, Chatsworth, CA). The methylation profiling of 22 liver cancer-related genes (*Ep300, Cdkn1b, Cdkn2a, Fhit, Pycard, Tnfrsf10d, Cdh1, Dlc1, Opcml, Reln, Ccnd2, Cdkn1a, Rassf1, Wt1, Socs1, Msh2, Msh3, Gstp1, Reln, E2f1, Runx3, Sfrp2*) was measured using EpiTect Methyl qPCR arrays according to the manufacturer’s instructions (Qiagen). This system does not require bisulfite conversion of DNA and relies on methylation-sensitive restriction enzymes. Briefly, 1 µg DNA was digested overnight at 37°C with the Methyl-Profiler DNA Methylation Enzyme Kit (Qiagen) containing methylation-sensitive and methylation-specific restriction enzymes, which digest unmethylated and methylated DNA, respectively. Then enzymatic activity was inactivated at 65°C for 20 min. The remaining DNA in each individual enzyme reaction was marked with SYBR Green then quantified using a 7500 real-time PCR system (Applied Biosystems, Foster City, CA, USA) using primers specific to one of the 22 liver cancer-related genes. The relative proportions of unmethylated and methylated DNA were then measured relative to the total DNA for each gene (measured when no restriction enzymes were added).

### Methylation-specific PCR

Genomic DNA was extracted from C57BL/6J mouse livers using the NucleoSpin TriPrep kit (Macherey-Nagel, Düren, Germany) according to the manufacturer’s instructions. 500 ng DNA was denatured using 0.3M NaOH (42°C, 30 min) then bisulfite modification was carried out by adding 1020 µL 40.5% sodium bisulfite, 60 µL 10 mM hydroquinone and 10 µL H_2_O, overlaying the mix with mineral oil and incubating at 55°C for 16 hours. The modified DNA was then purified using the Wizard DNA cleanup system (Promega) and resuspended in 100 µL TE. The purified DNA samples were then denatured (with NaOH) and precipitated with ethanol and stored at –20°C until use. The promoter methylation status of *p16*, *Mgmt* and *Dapk* genes was determined by methylation-specific PCR using primer sets described by Sharpless *et al.*, Yamada *et al.* and Mittag *et al,* respectively [34]–[36].

### Microarray analysis of miRNA levels

Total RNA was extracted from the livers of C57BL/6J mice (*n* = 3 per experimental condition) using TRIzol reagent (Invitrogen, Carlsbad, CA, USA). The profiling of miRNA expression levels was performed using the miRCURY LNA miRNA profiling service (Filgen). The quality of total RNA was checked using a Bioanalyzer 2100 (Agilent, Santa Clara, CA, USA), then RNA was labeled with Hy3 and hybridized to miRCURY LNA microRNA arrays 7^th^ generation (Exiqon, Vedbaek, Denmark). Slides were scanned using a laser scanner (Molecular Devices, Sunnyvale, CA, USA) and the resulting images were analyzed using Array-Pro v.4.5 (Media Cybernetics, Bethesda, MD, USA). Following background correction, quantile normalization was applied, so that the distribution remains the same across arrays [37]. Unsupervised hierarchical clustering and heatmap vizualisation of the samples for murine probes were performed using the CIMminer online software (http://discover.nci.nih.gov/cimminer/). Differentially expressed miRNA were identified by using the *t-*test within Significance Analysis of Microarrays (SAM) and median normalization [38]. The Diana miRPath tool v.2.1 [39] coupled with microT-CDS database was used to determine KEGG pathway [40] enrichment within the putative targets for the differentially expressed miRNAs. All array data have been submitted to the Gene Expression Omnibus (GEO) under the accession number GSE47956.

### Real-time PCR

For the validation of methylated genes expression, we used primers for *Socs1*, *Ep300*, *p16*, *Mgmt* and *Dapk* (QuantiTect Primer Assay, Qiagen); for the evaluation of miR-466e-5p gene targets expression, we used primers for *Zfp704*, *Cplx2* and *Cnot7* [41], [42]; for the validation of miRNA microarrays, we used primers for miR-466e-5p, miR-21-3p and miR-185-3p (MystiCq microRNA qPCR Assay Primer, Sigma-Aldrich). Mouse liver RNA was reverse-transcribed using the RT2 first strand kit (SABiosciences, Frederick, MD, USA) for gene expression experiments and MystiCq microRNA cDNA Synthesis Mix (Sigma-Aldrich) for miRNA expression experiments. Then quantitative real-time PCR reactions were performed in triplicate with an Applied Biosystems 7300 Real-Time PCR system (Life Technologies), using the RT2 SYBR Green PCR Master Mix (SABiosciences) for gene expression experiments and the MystiCq microRNA SYBR Green qPCR ReadyMix for miRNA expression experiments, according to the manufacturers’ instructions.

### Measurement of ROS levels

Intracellular levels of reactive oxygen species (ROS) in AML12 cells were measured using 5-(and-6)-chloromethyl-2′,7′-dichlorodihydrofluorescein diacetate, acetyl ester (CM-H2DCFDA, Molecular Probes, Eugene, OR, USA). Cells were plated in 12-well plates and loaded with pre-warmed PBS containing a mixture of oleic and palmitic acid for 12 hours, then 10 µM CM-H2DCFDA was added and cells were incubated for 40 minutes. Fluorescence intensities were measured using a SpectraMax M5 microplate reader (Molecular Devices, Sunnyvale, CA, USA) (excitation at 493 nm, emission at 520 nm). Unstained cells were used as negative control.

### Clonogenic assay

miR-466e inhibitor (Sigma-Aldrich) was transfected 48 hours before irradiation into AML12 cells using Lipofectamine RNAiMax reagent (Invitrogen), according to the manufacturer’s instructions (lipofectamine alone was added to control cells). Free fatty acids were added 24 hours before irradiation. After irradiation, cells treated or not with fatty acids were seeded at defined densities and incubated for 10–14 days then stained. Colonies with >50 cells were scored and surviving fractions were determined after correcting for the plating efficiency. Survival curve data were fitted to the linear-quadratic model [43] and are presented as the mean of at least three independent experiments.

## Results

### Diet-induced obesity does not modulate radiation-induced genotoxicity in the bone marrow

**Figure 1:**
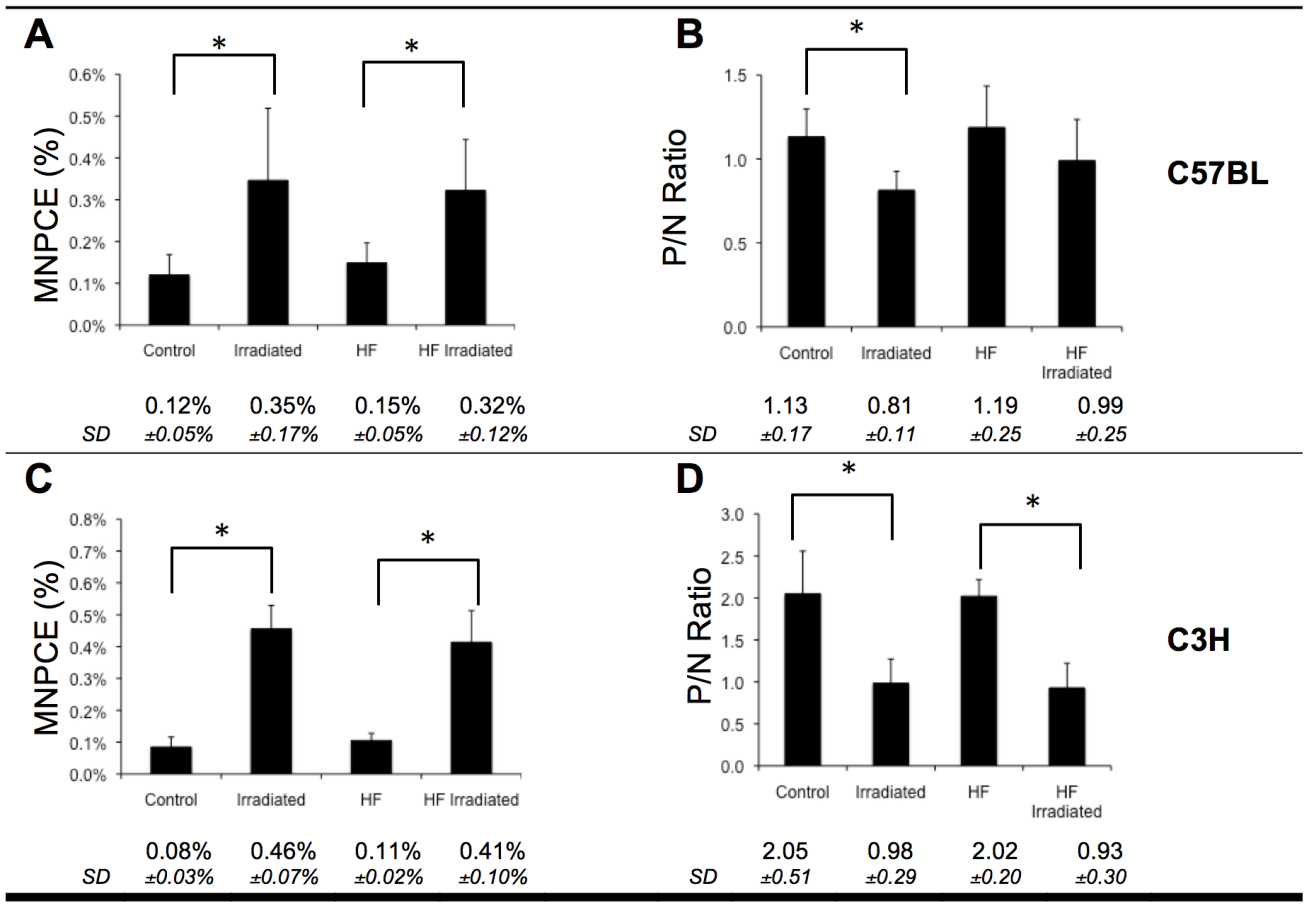
Bone marrow micronucleus test. A and C: Frequency of micronuclei in the bone marrow cells of C57BL/6J (A) and C3H (C) mice. B and D: ratio of polychromatic erythrocytes to normochromatic erythrocytes (P/N ratio) in the bone marrow of C57BL/6J (B) and C3H (D) mice. *p<0.05 compared to non-irradiated control (for irradiated mice fed a normal diet) or obese mice (for irradiated mice fed a HF diet).

Pre-diabetic obese C57BL/6J mice fed a high fat (HF) diet for several weeks kept gaining weight in our laboratory (Figure S1). C3H mice also accumulated additional weight when fed a HF diet, but did not exhibit metabolic syndromes. In order to evaluate whether DIO could influence radiation-induced genotoxicity in C57BL/6J and C3H mice, we measured bone marrow micronuclei frequencies after irradiation (Figure 1). Exposure to ionizing radiation resulted in increased MNPCE incidence regardless of diet in both mouse strains. No significant difference in MNPCE frequencies was observed between obese and non- obese animals, suggesting that DIO did not modulate radiation-induced genotoxicity.

**Figure 2:**
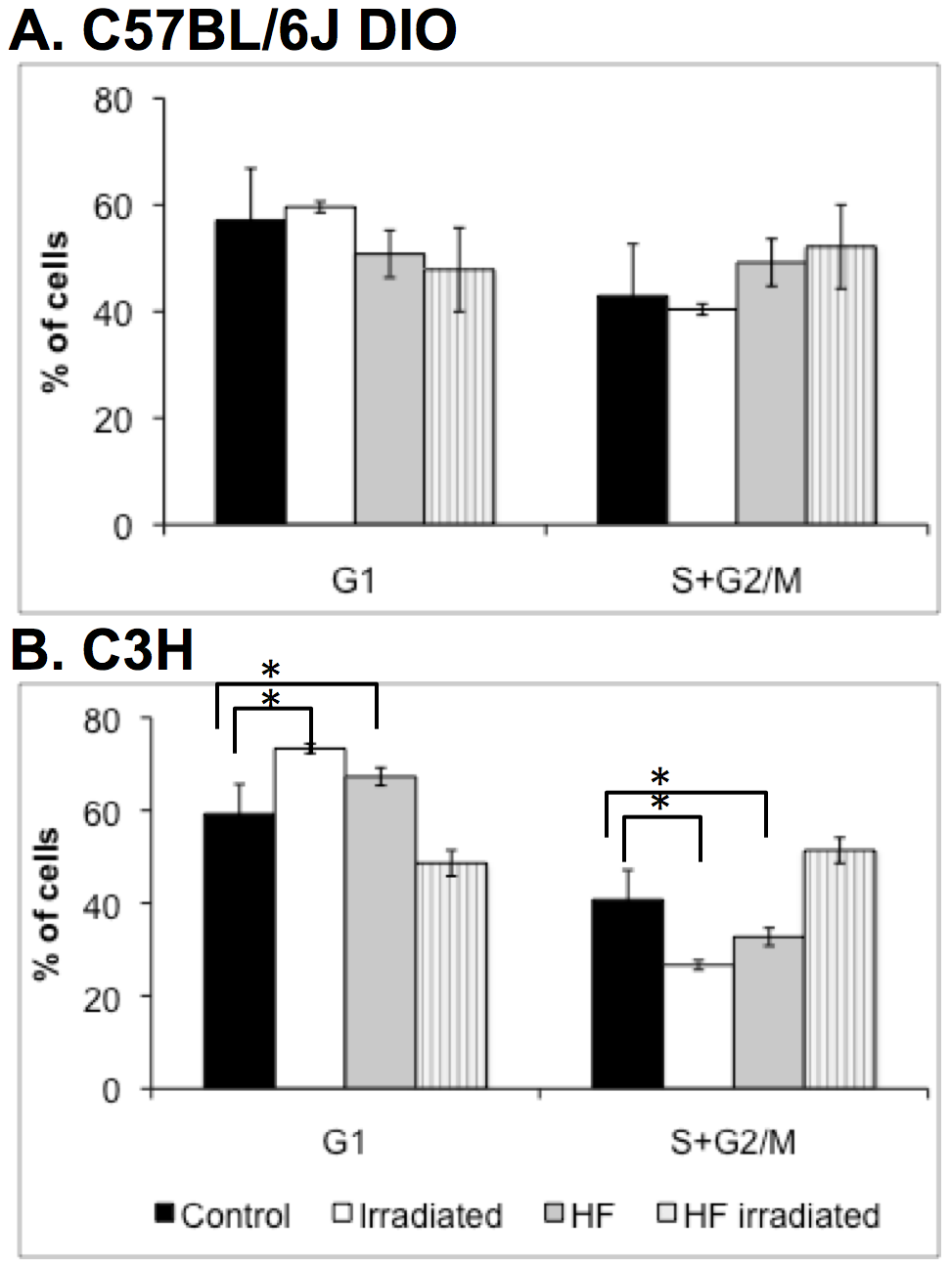
Cell cycle repartition of bone marrow TER-119^+^ nucleated cells. TER-119 was used as a lineage marker for erythroid cells. Analysis was performed in C57BL/6J (A) and C3H (B) mice using ModFit LT software. Results are the average of three independent experiments. Error bars represent standard deviation. *p<0.05 compared to control.

A significant decrease in P/N ratios was observed after irradiation in non-obese C57BL/6J and C3H mice (Figure 1B, D), indicative of cytotoxic damage or radiation-induced inhibition of proliferation. However, while exposure to ionizing radiation did also result in lower P/N ratio in obese C3H mice, C57BL/6J did not exhibit a significant radiation-induced decrease in P/N ratio. In order to determine whether the modulation of P/N ratio involved any modulation of erythroid cell proliferation, we studied the cell cycle repartition of nucleated erythroid progenitor and precursor cells in the bone marrow (Figure 2). TER-119+ cells are of the erythroid lineage and represent approximately 20% of the total cell number in the bone marrow. No significant difference in cell proliferation was observed in C57BL/6J mice after irradiation or depending on diet. In C3H mice, erythroid progenitors and precursor cells exhibited a lower entry into S/G2/M after irradiation or HF diet (p<0.01). The lower percentage of S/G2/M cells in irradiated animals suggested that the observed decreased P/N ratio resulted from an inhibition of cell proliferation of erythroid progenitors and precursors in response to radiation. However, surprisingly, C3H mice fed a HF diet did not show decreased numbers of erythroid cells in S/G2/M after irradiation, which may suggest that in obese mice, the cytotoxic effects of radiation affect mainly PCEs but not erythroid progenitors.

### Diet-induced obesity is associated with immediate modulation of promoter methylation in the liver

Even though DIO did not apparently modulate radiation-induced genotoxicity, we wondered whether immediate effects at the molecular or cellular level could be observed, which could ultimately influence radiation sensitivity and overall radiation risks in obese mice. Because liver is a major target organ for the metabolic syndromes resulting from DIO (including fatty liver disease), we then focused our investigations on effects in the liver.

**Figure 3:**
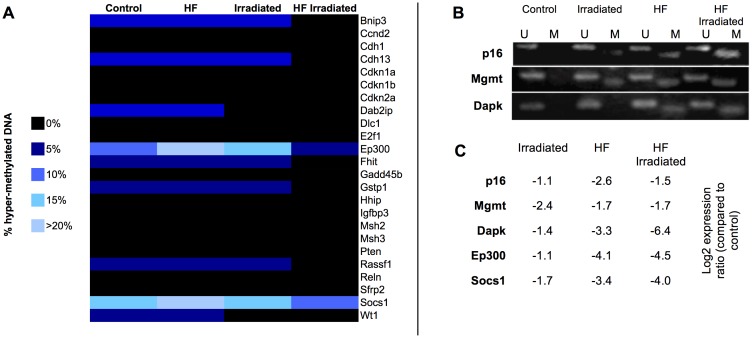
Gene promoter methylation in C57BL/6J mouse livers. A: Heatmap representing the promoter methylation status for 24 liver cancer-related genes in the livers of non-irradiated and irradiated C57BL/6J mice fed a normal or HF diet. B: Representative gels showing the methylation-specific PCR analysis of the promoter methylation status for *p16*, *Mgmt* and *Dapk* genes.

Promoter-specific hypermethylation is a frequent event in liver carcinogenesis [44]and is dependent on diet [45]. For this reason, we measured the methylation status of 27 gene promoters in the livers of C57BL/6J mice, using methylation PCR arrays and MSP (Figure 3). Methylation array provided us with methylation levels in the promoters of 24 liver cancer-related genes (Figure 3A). The percentages of highly-methylated DNA (as a fraction of total input DNA) never exceeded 10% for most of the genes, except E1A binding protein p300 (*Ep300*) and suppressor of cytokine signaling 1 (*Socs1*). It exceeded 20% for *Ep300* and *Socs1* genes in non-irradiated animals fed a HF diet, but it dropped to 7.8% and 12.4%, respectively, after irradiation. Our results suggested that *Ep300* expression might be inhibited in obese mice through promoter methylation, but not after irradiation. The results were less clear for *Socs1*, whose methylation levels were already elevated in control animals.

Previous investigations have described in various human and animal models the epigenetic regulation of promoter methylation for the tumor suppressors *p16* and *Dapk*, as well as for the O6-methylguanine-DNA methyltransferase *Mgmt* [46]–[49]. For this reason, we also measured by MSP the methylation status of these three genes in our model (Figure 3B). While no methylation was observed in control animals, HF diet triggered the methylation of *p16*, *Mgmt* and *Dapk* gene promoters (both in irradiated and in non-irradiated animals). *Mgmt* promoter was also methylated after irradiation in mice fed a normal diet. Interestingly, expression levels of *Ep300, Socs1, p16, Mgmt* and *Dapk* in obese and irradiated mice were significantly lower than in control mice (Figure 3C).

Overall our results suggest that the expression levels of several anti-tumour genes might be modulated by rapid promoter methylation in obese mice, with potential consequences in terms of radiation responses or carcinogenic risk (if sustained over the long-term).

### Diet-induced obesity influences micro-RNA regulations in the liver after irradiation

**Figure 4:**
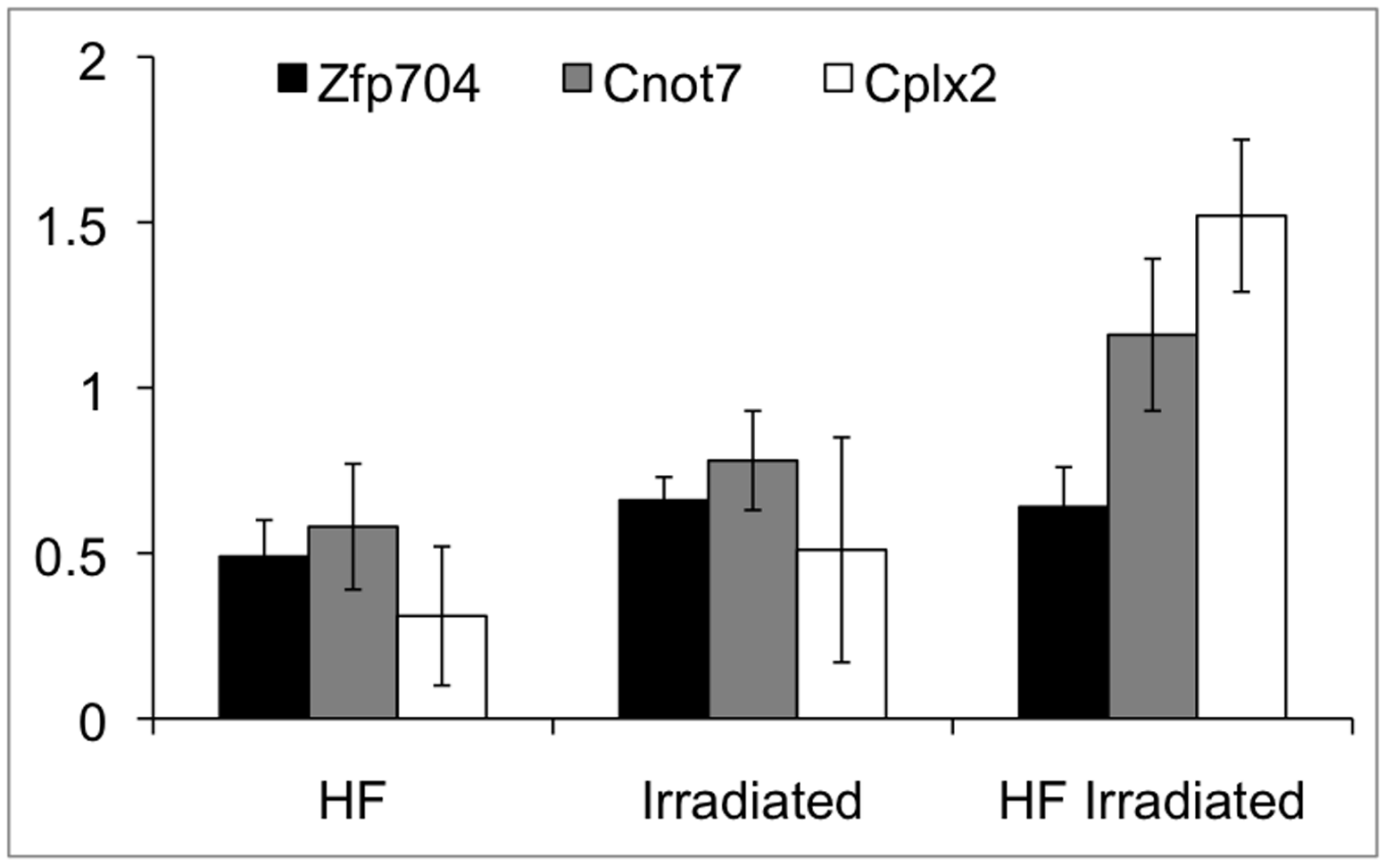
Expression levels of miR-466e-3p target genes. Expression levels of *Zfp704*, *Cnot7* and *Cplx2*, relative to control, were measured by quantitative real-time PCR. Results are the average of three independent experiments. Error bars represent standard deviation.

In addition to the regulation of gene expression by promoter methylation, diet-related epigenetic processes can also involve miRNAs. miRNA expression profiles in C57BL/6J mouse livers were analyzed by using locked nucleic acid (LNA) [50] miRNA arrays. Both HF diet and irradiation resulted in few consistent miRNA regulations among the sampled animals (three animals per experimental group) (Figure S2). Therefore SAM analysis was performed to compare miRNA expression levels between each experimental group (Table 1). SAM analysis provided a limited number of miRNAs that were consistently deregulated in every animal of each experimental group. Most of the observed modulations were upregulations. When compared to control animals (normal diet, non-irradiated), only one miRNA, miR-466e-5p, was upregulated in obese mice (no miRNA was downregulated). The putative targets genes of miR-466e-5p were identified by miRPath (using microT-CDS database) and the *Biosynthesis of fatty acids* KEGG pathway was identified by pathway enrichment analysis (p<0.01). After irradiation, 33 miRNAs were upregulated and 1 was downregulated (Table 1). 39 KEGG pathways (p<0.01) were found to be altered by miRPath (Table 2), including *Pathways in cancer, TGF-β signaling, MAPK signaling, Focal adhesion, Apoptosis* and *Wnt signaling pathway*, which are all known functions associated with radiation exposure [51]–[53]. On the contrary, in irradiated obese mice, only one miRNA (miR-3961) was upregulated compared to control and no miRNA was modulated compared to non-irradiated obese mice: the above-mentioned radiation-induced miRNA regulations were not observed in obese mice. We verified by real-time quantitative PCR the expression levels of miR-466e-3p, miR-185-3p and miR-21-3p; both miR-466-e-3p and miR-21-3p were upregulated after high-fat diet and irradiation, respectively (Table S1). To evaluate the biological significance of miR-466e-3p upregulation in obese mice, we measured by real-time quantitative PCR the expression levels of three target genes: *Zfp704*, *Cnot7* and *Cplx2* (Figure 4). These three genes were downregulated in mice fed a HF diet.

**Table 1:** miRNA regulations in the livers of non-irradiated and irradiated C57BL/6J mice fed a normal or HF diet, determined using Significance Analysis of Microarrays (SAM).

HF				Irradiated				HF, irradiated			
Upregulated		Downregulated		Upregulated		Downregulated		Upregulated		Downregulated	
	FC		FC		FC		FC		FC		FC
*vs Control*		*vs Control*		*vs Control*		*vs Control*		*vs Control*		*vs Control*	
miR-466e-5p	1.7	none		miR-1187	2.3	miR-5616-5p	−2.7	miR-3961	1.5	none	
				miR-185-3p	2.7						
				miR-1897-5p	2.1			*vs HF*		*vs HF*	
				miR-1900	1.8			none		none	
				miR-1935	2						
				miR-1947-3p	2.3						
				miR-1971	2.5						
				miR-21-3p	2.9						
				miR-3082-5p	2.2						
				miR-3098-3p	2.3						
				miR-3100-3p	2.5						
				miR-32-3p	2.1						
				miR-346-3p	2.5						
				miR-3961	1.5						
				miR-466f	1.9						
				miR-466f-3p	2.3						
				miR-466i-3p	2.5						
				miR-466i-5p	2.2						
				miR-466q	2.4						
				miR-467e-3p	2.2						
				miR-467f	2.5						
				miR-467g	2.3						
				miR-5113	2.3						
				miR-574-5p	2.1						
				miR-669a-3-3p	2.2						
				miR-669c-5p	2.2						
				miR-669e-3p	2.4						
				miR-669f-3p	2.4						
				miR-669k-5p	1.9						
				miR-669l-5p	2.1						
				miR-669n	2.1						
				miR-669o-5p	1.8						
				miR-744-5p	2						

Fold-changes (FCs) of miRNA expression were measured as compared to non-irradiated control mice (*vs Control*) or to non-irradiated mice fed a HF diet (*vs HF).* If the expression ratios were >1, then FCs were equal to expression ratios. If the expression ratios were <1, then FCs were equal to the opposite of expression ratios.

**Table 2:** Altered KEGG pathways (p<0.01) associated with the putative target genes of the miRNAs regulated by ionizing radiation exposure in mice fed a normal diet.

	KEGG pathway	Nb of genes	Nb of miRNAs
1	Pathways in cancer	122	28
2	TGF-beta signaling pathway	36	23
3	MAPK signaling pathway	98	29
4	Focal adhesion	76	27
5	Glycosaminoglycan biosynthesis - heparan sulfate	7	12
6	Regulation of actin cytoskeleton	79	27
7	Colorectal cancer	28	24
8	Melanoma	34	24
9	ErbB signaling pathway	34	26
10	Renal cell carcinoma	30	25
11	Endometrial cancer	24	24
12	Apoptosis	32	25
13	Glioma	25	22
14	Prostate cancer	38	25
15	Adherens junction	32	23
16	Pancreatic cancer	30	25
17	Calcium signaling pathway	60	27
18	T cell receptor signaling pathway	43	27
19	Non-small cell lung cancer	21	22
20	Melanogenesis	36	24
21	Long-term depression	23	22
22	Osteoclast differentiation	44	26
23	Toxoplasmosis	40	23
24	Acute myeloid leukemia	24	23
25	Axon guidance	46	23
26	Gap junction	33	23
27	Wnt signaling pathway	55	25
28	Neurotrophin signaling pathway	48	30
29	Hedgehog signaling pathway	23	19
30	Endocrine and other factor-regulated calcium reabsorption	21	21
31	Lysine degradation	14	16
32	mTOR signaling pathway	22	21
33	Ubiquitin mediated proteolysis	45	26
34	Gastric acid secretion	29	23
35	SNARE interactions in vesicular transport	14	16
36	ECM-receptor interaction	25	18
37	N-Glycan biosynthesis	15	17
38	Bacterial invasion of epithelial cells	26	26
39	Glycosphingolipid biosynthesis - lacto and neolacto series	9	15

### Free fatty acids generate oxidative stress and sensitize liver cells to ionizing radiation

**Figure 5:**
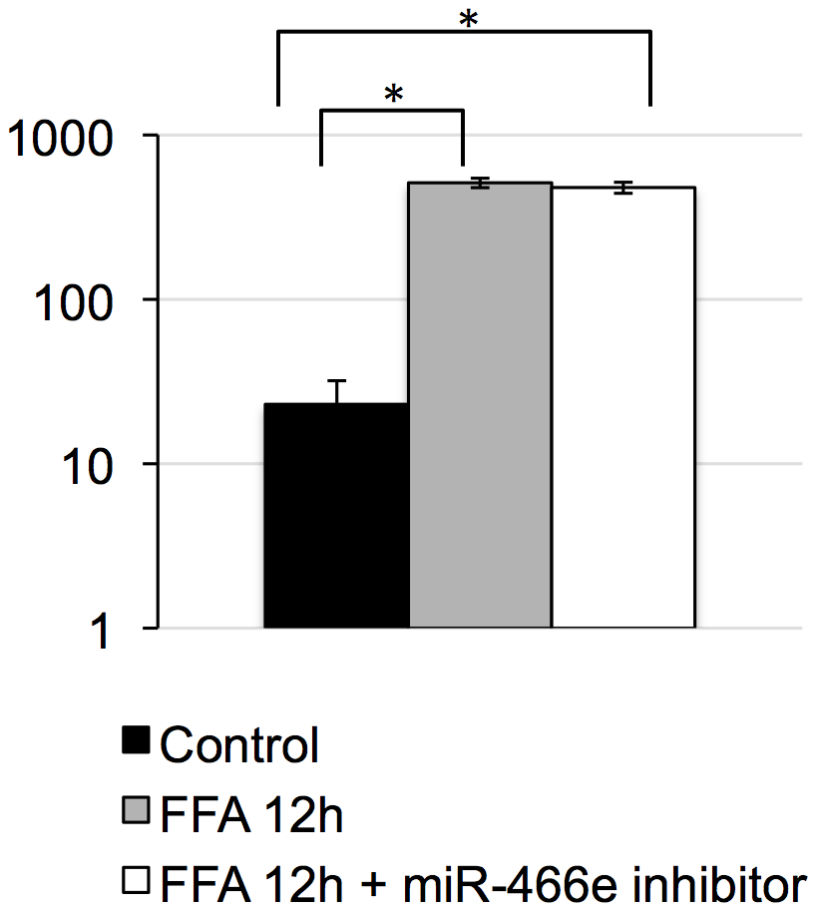
ROS levels in AML12 cells. ROS levels were measured after treatment with free fatty acids (FFAs) oleic acid and palmitic acid and with miR-466e inhibitor. Results are the average of three independent experiments. Error bars represent standard deviation. Asterisks denote significant differences (*t-*test, *p<0.01).

**Figure 6:**
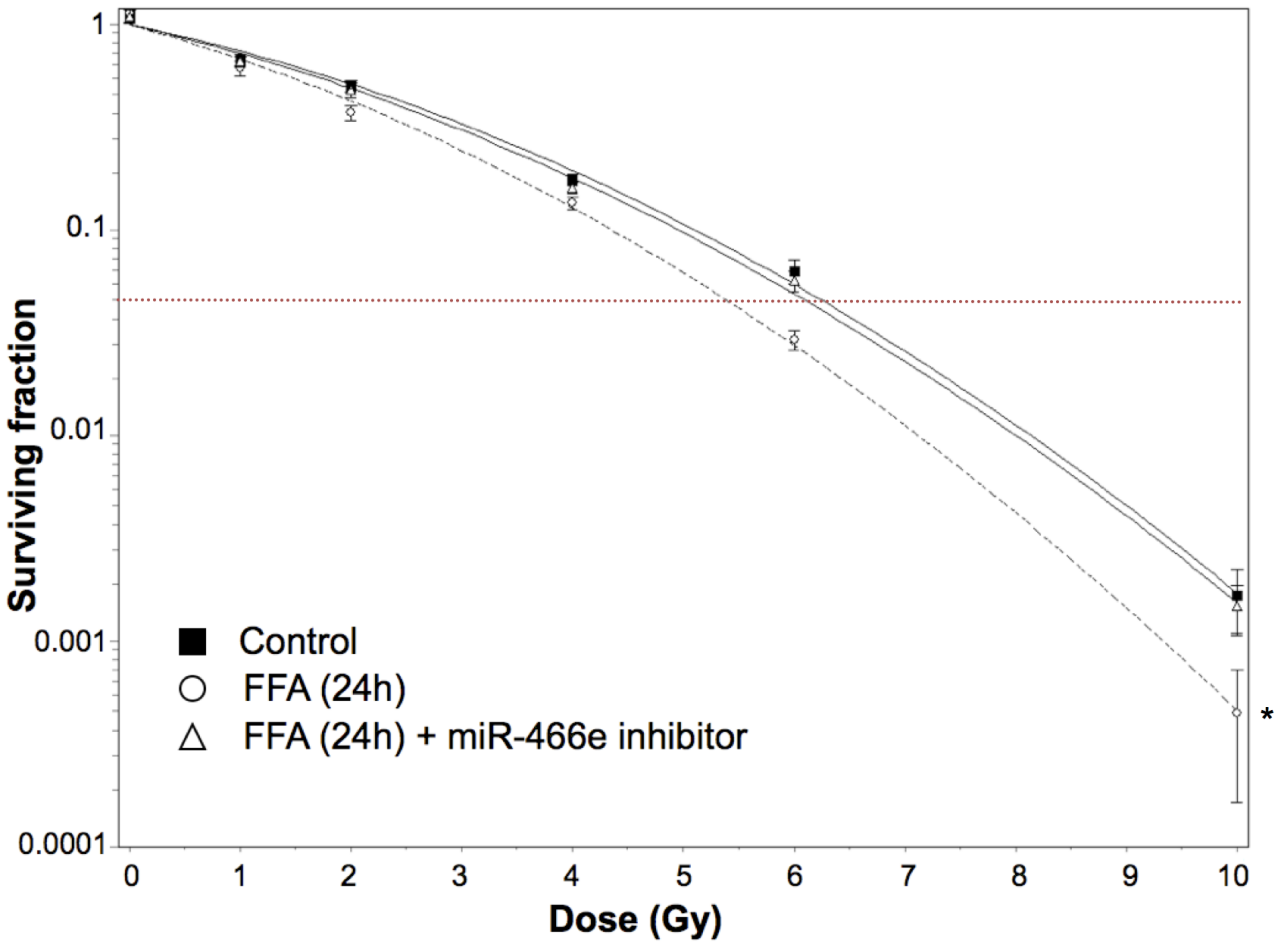
Dose-response curves for clonogenic survival of AML12 cells. AML12 cells were treated for 24 hours with free fatty acids (FFAs: oleic acid, palmitic acid) alone (circles, dotted line) or both with miR-466e inhibitor and FFAs (triangles, continuous line) then exposed to various doses of ionizing radiation. Lipofectamine alone was added to control cells (squares, continuous line). Lines represented fitted curves according to linear quadratic regression. The red dotted line indicates 5% clonogenic survival (DL95). Results are the average of at least three independent experiments. Error bars represent standard deviation. Statistical significance of the difference between dose-response curves was performed using one-way Analysis of Variance (one-way ANOVA) with Bonferroni correction for pairwise group comparisons. *p<0.01 compared to control.

In order to mimic *in vitro* the effects of obesity on radiation responses in the mouse liver, we treated AML12 murine liver cells with free fatty acids (FFAs: palmitic acid and oleic acid) for 12 to 24 hours. Twelve hours after FFA administration, ROS levels increased significantly, reminiscing of chronic oxidative stress in C57BL6 DIO mouse livers; the addition of miR-466e inhibitor did not influence ROS levels (Figure 5). The reproductive clonogenic viability of AML12 cells 24 hours after FFA treatment was determined using the clonogenic assay (Figure 6). The dose that gave 5% mean clonogenic survival (DL95) was lower after FFA treatment (FFA: 5.27 Gy, Control: 6.12 Gy), indicating that FFAs sensitized cells to ionizing radiation. However, FFA-triggered sensitization was counteracted by the administration of miR-466e inhibitor, suggesting that miR-466e expression might influence radiation-sensitivity.

## Discussion

Given the importance of diet in the development of obesity-associated metabolic syndromes, a significant amount of interest has been given to the molecular analysis of metabolic disruptions in obesity. In particular, there is growing evidence showing that diet can strongly influence epigenetic processes [21]. For this reason, we wondered whether metabolic disturbances associated with obesity were susceptible to influence radiation sensitivity.

In order to understand the combinational effects of DIO and ionizing radiation in mice, we exposed C57BL/6J and C3H mice to fractionated doses of X-rays. DIO mice exhibit excessive body weight, body fat accumulation, prediabetes type 2 and metabolic syndrome. C3H mice were also studied because they are comparatively resistant to metabolic syndrome and atherosclerosis [54]. High 60% fat (HF) chow provided an energy density of 5.24 kcal/g (compared to 3.85 kcal/g for the control 10% fat chow) and mice fed a HF diet exhibited an accumulation of body fat and became obese, while mice fed a control diet did not.

There are ample amounts of evidence describing increased bone marrow MN frequency after radiation exposure in mice [55]–[59]. On the contrary, HF diet did not modulate bone marrow MN frequency both in non-irradiated and irradiated mice, in contradiction with human data describing a correlation between peripheral blood MN frequency and pathologies associated with metabolic syndrome and obesity [60]. The P/N ratio was higher in C3H than in C57BL/6J control mice, suggesting that these mice have a higher rate of erythropoiesis. Whereas no significant proliferation changes were observed in C57BL/6J mice, both exposure to radiation and HF diet resulted in lower erythroid precursors cell proliferation in C3H mice. In obese animals, it was suggested that bone marrow adipogenesis may impair erythropoiesis, resulting in anemia [61]. Rapidly cycling early erythroid progenitors are highly radiosensitive and encounter cell cycle damage checkpoints and apoptosis in response to radiation, compared to more mature erythroid cells [62]. Interestingly, this inhibition of proliferation was not observed in obese C3H mice after irradiation, suggesting that the progenitor cells might not respond to radiation exposure the same way in obese mice than in control mice, or that in response to both HF diet and radiation exposure, erythropoiesis might be stimulated. Additional investigations are necessary to understand the underlying mechanisms.

Obesity and obesity-related complications play a significant role in the pathogenesis of liver diseases and liver cancer [63], [64]; for this reason, we focused our analysis on the interplay between radiation-responses and DIO in the mouse liver. Because DNA methylation is known to play a crucial role in carcinogenesis by regulating the expression of tumor suppressor or DNA repair genes [65], we measured the methylation profiles of several genes associated with liver cancer. While short-term modulation of promoter methylation was previously observed in C57BL/6J mice exposed to acute or chronic X-ray irradiation [66], only the *Mgmt* gene presented a methylated form after irradiation in animals fed a normal diet. *Mgmt* plays a major role in protecting cells from the genotoxic and carcinogenic effects of methylating mutagens [67]. Increased methylation of the *Mgmt* gene promoter might result in lower *Mgmt* gene expression levels, in contradiction with previous observations showing that genotoxic stress (including ionizing radiation) can induce *Mgmt* expression in rat liver cells [68], [69]. However, MSP does only provide qualitative results at best and additional investigations are required to measure *Mgmt* gene expression levels in our model. Our results also suggested that DIO was associated with increased hypermethylation of *Ep300*, *p16* and *Dapk* gene promoters. Inactivation of tumor suppressor *p16* by aberrant methylation is frequently observed in carcinomas and precancerous lesions of various organs [48], [49]. Similarly, aberrant methylation of the apoptosis-related *Dapk* promoter is extremely common in cancers [34], [46]. These tumor suppressor genes exhibited promoter hypermethylation only in obese animals, suggesting potential effects in terms of radiation response and carcinogenesis. Expression levels of *p16, Mgmt*, *Dapk, Ep300* and *Socs1* were systematically lower in obese and irradiated animals, suggesting that promoter methylation contributes to gene repression in these animals.

In a similar model of DIO in C57BL/6J mice, microarray analysis revealed that the expression of 97 genes was significantly modulated in the livers of mice fed a HF diet, compared to mice fed a normal-fat diet [70]. These genes were mainly involved in metabolism, stress defense mechanisms (such as protection against oxidative damage) and inflammatory processes. Other microarray analyses revealed that a number of genes are deregulated in response to ionizing radiation [71], [72], including at very low dose-rates [73], [74]. Because gene expression can be modulated by miRNAs, we studied diet- and radiation-associated miRNA regulations in C57BL/6J mouse livers.

Recent studies have described obesity-related miRNA regulations in mouse and human models. These miRNAs were involved in various processes such as adipocyte differentiation, metabolic integration, insulin resistance and appetite regulation [75]. HF diet-associated miRNA up- (miR-22, miR-342-3p, miR-142-3p and others) and down-regulations (miR-200b, miR-200c, miR-204 and others) were observed in the adipose tissues of C57BL/6J mice [76]. In the liver, there is convincing evidence that miR-122 is involved in cholesterol and lipid metabolism, and the silencing of miR-122 has resulted in decreased cholesterol levels in mice and monkeys [77]–[79]. We did not observe any significant modulation of miR-122 in the livers of obese and/or irradiated mice. However, using SAM analysis, we identified a new HF diet-associated miRNA upregulation (miR-466e); additionally, the modulation of miR-709 expression was under the fold-change cut-off value of 1.5 but was potentially associated with HF diet as suggested by SAM analysis. miR-709 was recently described as a tumor-suppressor miRNA targeting Myc, Akt and Ras, which participates in the regulation of apoptosis through the miR-15a/miR-16-1 pathway [80], [81]. HF diet resulted in the down-regulation of three putative miR-466e target genes (*Zfp704*, *Cnot7*, *Cplx2*), and it is highly likely that a number of genes are regulated in response to miR-466e up-regulation. A functional analysis of the putative target genes of miR-709 and miR-466e suggested that these miRNAs might be involved in lipid metabolism. miR-466e gene is included in the *Chromosome 2 miRNA cluster* (C2MC, or *Sfmbt2 cluster*), a mouse-specific large miRNA cluster located in intron 10 of the *Sfmbt2* gene. C2MC contains 71 closely related miRNA genes modulated in response to cellular stress (nutrition deprivation, hyperglycemia, hypertonic stress, oxidative stress, xenobiotic stress, aging…) and involved in cell proliferation and apoptosis, cell fate decision and immune response [82]–[85].

For this reason, we hypothesized that miR-466e might also be involved in stress response in the mouse liver, and we evaluated *in vitro* the effects of free fatty acid (FFA) administration and the potential role of miR-466e in the response of AML12 mouse liver cells to radiation. Although miR-466e inhibition did not modulate FFA-induced ROS levels, it counteracted the radiosensitization of AML12 cells by FFAs. Interestingly, it was recently shown that oxidative stress triggered acetylation in the miR-466h-5p promoter region, leading to the activation of this miRNA closely related to miR-466e [82]. Further investigations are needed to understand whether miR-466e is regulated through similar mechanisms, and to decipher the biological function of miR-466e.

We observed that a number of miRNAs were upregulated after irradiation in mice fed a normal diet. A number of other studies have recently identified miRNAs associated with radiation responses [86], [87]. In particular, miR-21, which targets many genes in the apoptotic pathway, was consistently seen to be upregulated after exposure to ionizing radiation in various models [88]–[91]; miR-21 was also upregulated in radiation-induced thymic lymphoma in BALB/c mice [92]. Many of the deregulated miRNAs in our study are not yet fully characterized and additional investigations will likely provide more insights into their potential involvement in radiation responses. Many of the molecular pathways identified by the miRPath tool in irradiated mice fed a normal diet were known to be associated with radiation responses and/or cancer, such as the TGF-β [93], MAPK [94], ErbB [95] and Wnt [53] signaling pathways, apoptosis, etc. Interestingly, these radiation-induced miRNA regulations were not observed in irradiated obese mice. This could not be explained by a previous up-regulation associated with HF diet, since only one miRNA was deregulated in obese mice.

We therefore suggest that the molecular pathways normally involved in radiation responses are disrupted in obese mice. The question remains whether those might influence long-term effects, such as radiation-induced carcinogenesis. It is highly possible that because obese animals experience chronic oxidative stress (as reported by others and suggested by our immunohistochemistry results) [96], they are further sensitized to ionizing radiation risks. 8-OHdGs in CpG sequences were shown to inhibit the methylation of neighboring cytosine residues, resulting in DNA hypomethylation [97]. However, obesity was associated in our model with increased promoter methylation for several genes.

A substantial body of evidence shows that oxidative stress is a major contributor to the deleterious effects of obesity [98]. The expansion of abdominal fat results in elevated levels of free fatty acids (FAAs) [99], [100], which stimulate reactive oxygen species (ROS) production [98] and induce hepatic insulin resistance as well as increased glucose production. Furthermore, decreased antioxidant capacity levels and increased DNA damage were described in relationship with obesity-induced chronic oxidative stress [96]. The disruption of genetic and epigenetic mechanisms by obesity-induced chronic oxidative stress might result in a deficient response to ionizing radiation. For example, it was shown that the deregulation of protein kinase C (PKC) signaling, an early responder to ionizing radiation [101], is a crucial event in obesity-related metabolic syndrome and oxidative stress [102], [103]. Irradiation in obese animals might thus result in increased cancer risk. Furthermore, miRNA regulations (such as the upregulation of miR-466e) are likely to play a significant role in regulating radiation response.

Overall, it is necessary to better understand the crosstalk between obesity-related metabolic syndrome and radiation responses, both at low and high dose ranges, in order to better evaluate the risks and effects of ionizing radiation resulting from natural or medical exposures, or to develop specific radiation-therapy regimens adapted to each patient characteristics.

## Supporting Information

Figure S1 **Time-course of weight gain in mice.** C57BL/6J (left) and C3H (right) mice were fed a normal (black symbols) and a HF diet (colored symbols).
(PDF)

Figure S2 **Heatmap of miRNA expression in the livers of obese and/or irradiated C57BL/6J DIO mice.** Unsupervised hierarchical clustering of 1157 mouse miRNAs (100% of known mouse miRNAs) was performed. C: control (mice No. 116, 117, 118); HF: HF diet (mice No. 106, 107, 108); R: irradiated (mice No. 111, 112, 113); HFR: HF diet, irradiated (mice No. 101, 102, 103).
(PDF)

Table S1 **Real-time PCR verification of miRNA expression.** Expression ratios (compared to control) of miR-466e-3p, miR-185-3p and miR-21-3p were measured by RT-PCR. Green values indicate miRNAs identified as upregulated in the SAM microarray analysis.
(PDF)
