# Supplementary material for: Diet-Induced Obesity Modulates Epigenetic Responses to Ionizing Radiation in Mice
Source: PLoS One. 2014 Aug 29;9(8):e106277. doi: 10.1371/journal.pone.0106277 (PMC4149562; doi:10.1371/journal.pone.0106277)

Figure S1
Time-course of weight gain in mice. C57BL/6J (left) and C3H (right) mice were fed a normal (black symbols) and a HF diet (colored symbols).

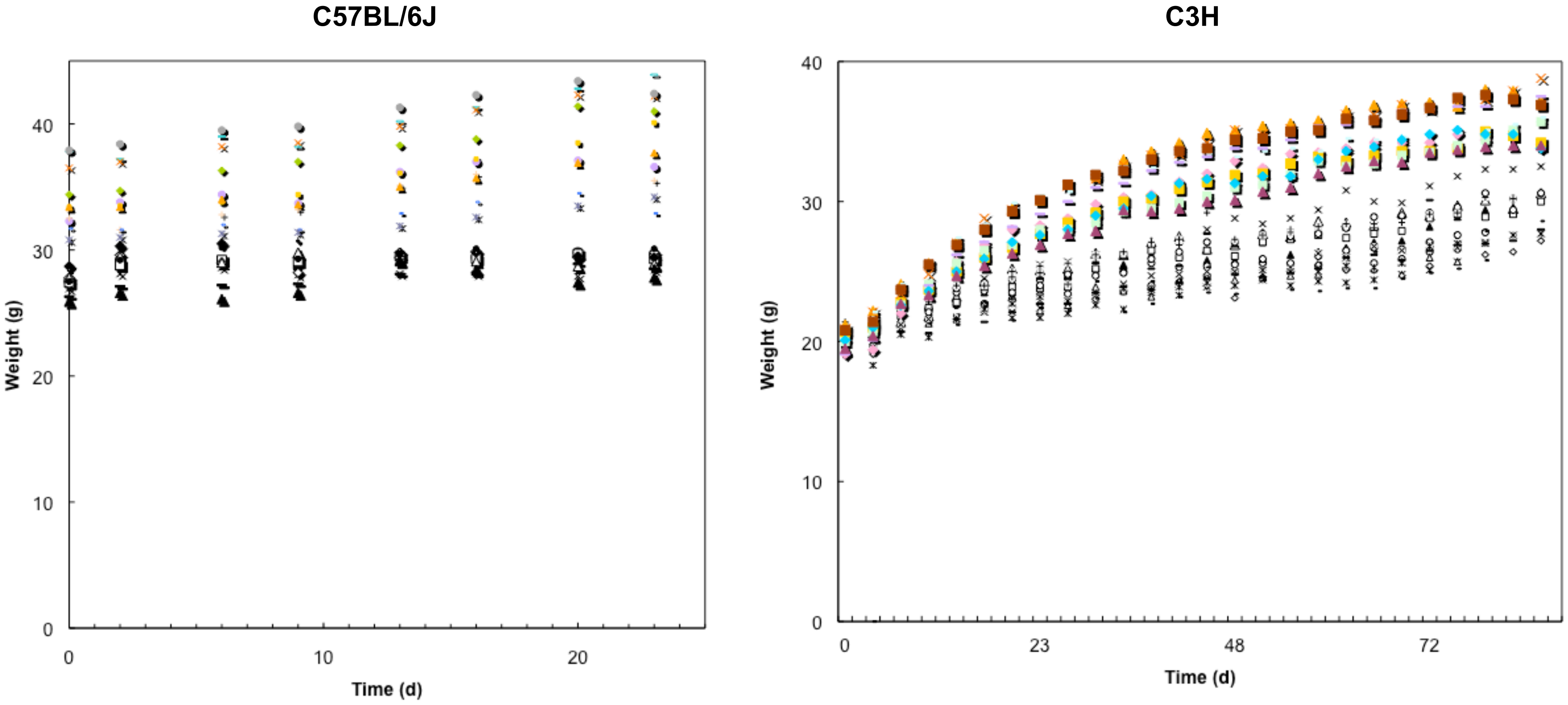

Supplement: Figure S1 — Time-course of weight gain in mice. C57BL/6J (left) and C3H (right) mice were fed a normal (black symbols) and a HF diet (colored symbols). (PDF) [file pone.0106277.s001.pdf]
