# Supplementary material for: Diet-Induced Obesity Modulates Epigenetic Responses to Ionizing Radiation in Mice
Source: PLoS One. 2014 Aug 29;9(8):e106277. doi: 10.1371/journal.pone.0106277 (PMC4149562; doi:10.1371/journal.pone.0106277)

Figure S2

Heatmap of miRNA expression in the livers of obese and/or irradiated C57BL/6J DIO mice. Unsupervised hierarchical clustering of 1157 mouse miRNAs (100% of known mouse miRNAs) was performed. *C*: control (mice No. 116, 117, 118) ; *HF*: HF diet (mice No. 106, 107, 108) ; *R*: irradiated (mice No. 111, 112, 113) ; *HFR*: HF diet, irradiated (mice No. 101, 102, 103).

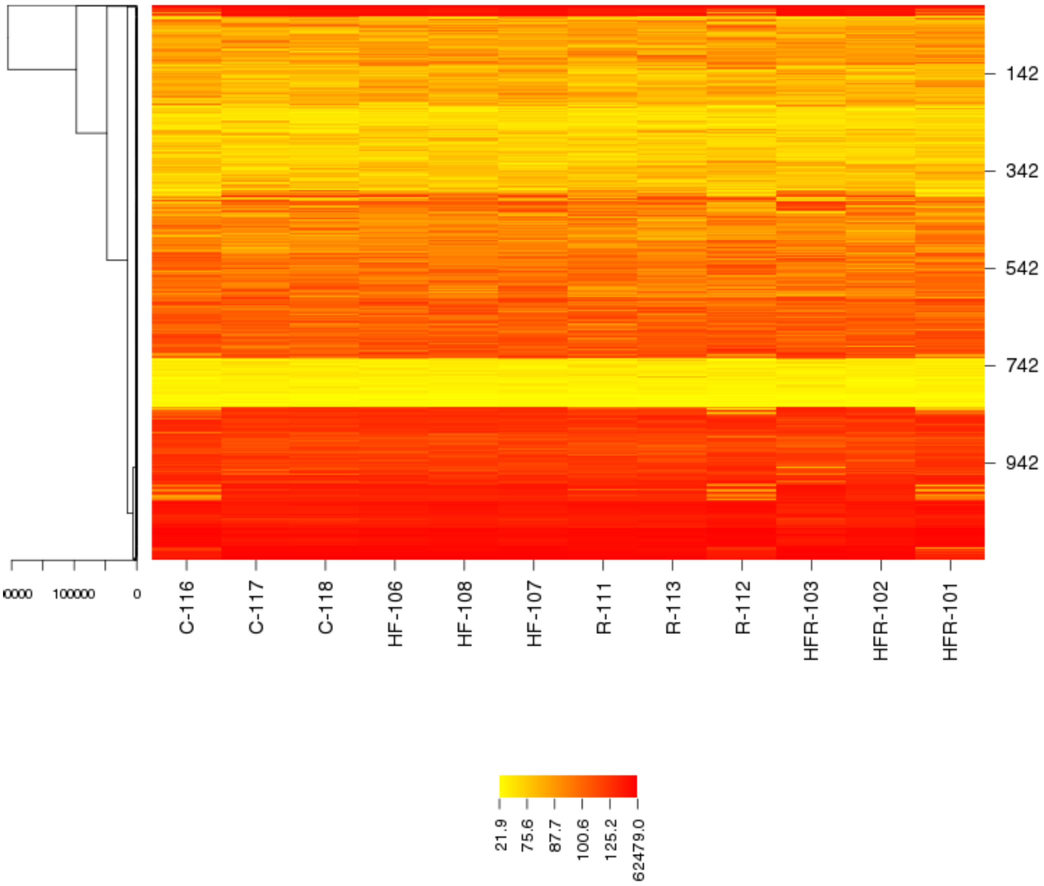

Supplement: Figure S2 — Heatmap of miRNA expression in the livers of obese and/or irradiated C57BL/6J DIO mice. Unsupervised hierarchical clustering of 1157 mouse miRNAs (100% of known mouse miRNAs) was performed. C: control (mice No. 116, 117, 118); HF: HF diet (mice No. 106, 107, 108); R: irradiated (mice No. 111, 112, 113); HFR: HF diet, irradiated (mice No. 101, 102, 103). (PDF) [file pone.0106277.s002.pdf]
