# Supplementary material for: Diet-Induced Obesity Modulates Epigenetic Responses to Ionizing Radiation in Mice
Source: PLoS One. 2014 Aug 29;9(8):e106277. doi: 10.1371/journal.pone.0106277 (PMC4149562; doi:10.1371/journal.pone.0106277)
Supplement: Table S1 — Real-time PCR verification of miRNA expression. Expression ratios (compared to control) of miR-466e-3p, miR-185-3p and miR-21-3p were measured by RT-PCR. Green values indicate miRNAs identified as upregulated in the SAM microarray analysis. (PDF) [file pone.0106277.s003.pdf]

Table S1

Real-time PCR verification of miRNA expression. Expression ratios (compared to control) of miR-466e-3p, miR-185-3p and miR-21-3p were measured by RT-PCR. Green values indicate miRNAs identified as upregulated in the SAM microarray analysis.

	**High-fat diet**	**Irradiated**	**High-fat diet, irradiated**
**miR-466e-3p**	4.22 (± 1.25)	6.21 (± 3.11)	0.41 (± 0.13)
**miR-185-3p**	1.71 (± 0.75)	1.33 (± 0.90)	0.17 (± 0.03)
**miR-21-3p**	0.75 (± 0.35)	4.41 (± 1.90)	0.50 (± 0.16)
